# Which ultrasound transducer type is best for diagnosing pneumothorax?

**DOI:** 10.1186/s13089-018-0109-0

**Published:** 2018-10-22

**Authors:** R. Ketelaars, E. Gülpinar, T. Roes, M. Kuut, G. J. van Geffen

**Affiliations:** 1 Department of Anesthesiology, Pain and Palliative Medicine, Radboud University Medical Center, Radboud Institute for Health Sciences, Geert Grooteplein-Zuid 10, 6525 GA Nijmegen, The Netherlands; 2 Helicopter Emergency Medical Service-Lifeliner 3, Radboud University Medical Center, Radboud Institute for Health Sciences, Geert Grooteplein-Zuid 10, 6525 GA Nijmegen, The Netherlands

**Keywords:** Ultrasonography, Transducer, Pneumothorax, Emergency medical services

## Abstract

**Background:**

An accurate physical examination is essential in the care of critically ill and injured patients. However, to diagnose or exclude a pneumothorax, chest auscultation is unreliable compared to lung ultrasonography. In the dynamic prehospital environment, it is desirable to have the best possible ultrasound transducer readily available. The objective is to assess the difference between a linear-array, curved-array, and phased-array ultrasound transducer in the assessment for pneumothorax and to determine which is best.

**Methods:**

In this double-blinded, cross-sectional, observational study, 15 observers, experienced in lung ultrasonography, each assessed 66 blinded ultrasound video clips of either normal ventilation or pneumothorax that were recorded with three types of ultrasound transducers. The clips were recorded in 11 adult patients that underwent thoracoscopic lung surgery immediately before and after the surgeon opened the thorax. The diagnostic accuracy of the three transducers, elapsed time until a diagnosis was made, and the perceived image quality was recorded.

**Results:**

In total, 15 observers assessed 990 ultrasound video clips. The overall sensitivity and specificity were 98.2% and 97.2%, relatively. No significant difference was found in the diagnostic performance between transducers. A diagnosis was made slightly faster in the linear-array transducer clips, compared to the phased-array transducer (*p* = .031). For the linear-, curved-, and phased-array transducer, the image quality was rated at a median (interquartile range [IQR]) of 4 (IQR 3–4), 3 (IQR 2–4), and 2 (IQR 1–2), relatively. Between the transducers, the difference in image quality was significant (*p* < .0001).

**Conclusions:**

There was no difference in diagnostic performance of the three transducers. Based on image quality, the linear-array transducer might be preferred for (prehospital) lung ultrasonography for the diagnosis of pneumothorax.

## Background

In the critically ill and injured patient, an accurate physical examination is essential in the care of the patient. However, auscultating for breath sounds in a respiratory distressed patient is often difficult or even impossible, especially in a noisy accident scene, or patient compartment of a (moving) ambulance or helicopter.

The sensitivity of auscultation for the diagnosis of hemothorax, hemopneumothorax, and pneumothorax is only 58–66% [1–3]. Unilateral decreased or absent breath sounds are often interpreted as a pneumothorax. However, conditions such as splinting from rib pain, lung contusion, atelectasis, pneumonia, pleural effusion, and tumor growth may account for the same abnormal auscultation.

Lung ultrasonography (US) for the diagnosis of pneumothorax was first described in 1986 [4]. It may rule-in pneumothorax with a sensitivity ranging from 81 to 98% and rule it out with a specificity approaching 99–100% [5–7]. Additionally, pleural effusion, lung contusion, and atelectasis may be detected [7]. It has even been suggested that US might 1 day replace the stethoscope [8, 9].

US is feasible in the prehospital setting including inside ground ambulances and a helicopter emergency medical service (HEMS) [10–12]. Similar to most diagnostic and therapeutic procedures, US requires training and regular practice. Time pressure and limited working space are additional challenges [12]. To facilitate the best possible images, it is important that optimally set-up US equipment is readily available. In an optimal configuration, the most suitable transducer is connected to the US machine.

Lung US can be performed with high-frequency linear-array, curved-array, or phased-array transducers. However, it is not known which one is preferable and provides the best images.

We hypothesized that a linear-array transducer is the optimal transducer for the appreciation of the pleural line for diagnosing pneumothorax. The aim of the study is to compare three transducer types on diagnostic accuracy, speed of the diagnosis, and image quality in the assessment for pneumothorax.

## Methods

We performed a double-blinded, cross-sectional, observational study to compare three types of ultrasound transducers for the diagnosis of two conditions: normal ventilation, and pneumothorax. Ethical approval was obtained from the institutional ethics review board of the Radboud university medical center, Nijmegen. Written informed consent was asked and obtained from every patient and from every observer.

At the preoperative outpatient evaluation clinic of the Radboud university medical center, Nijmegen, the Netherlands, from September to October 2017, we recruited a consecutive series of eleven eligible patients that were scheduled for video-assisted thoracoscopic surgery (VATS) for pulmonary, mainly neoplastic, pathology. The inclusion criteria were a body mass index < 30 kg m^−2^ and the absence of pathology of the chest wall, visceral or parietal pleura.

Lung US is a valuable test for the detection or exclusion of a pneumothorax [13, 14]. A US transducer is positioned on the chest wall perpendicular to two adjacent ribs. Between the acoustic shadows of two ribs, a hyperechoic line is visible representing the interface of the parietal and visceral pleura. With normal ventilation, lung sliding is observed as a to-and-fro movement at the pleural line as a result of the sliding of the visceral pleura against the inner chest wall. B-lines may be observed as hyperechoic lines radiating down from the pleural line. Their presence excludes pneumothorax (at the transducer position). Horizontal repetitions of the pleural line appearing below at multiples of the skin-pleural line distance are called A-lines. Their appearance is more prominent in the presence of a pneumothorax when B-lines are absent and no longer obscuring the A-lines.

We used a portable X-Porte ultrasound system (Fujifilm SonoSite Inc., Bothell, WA, USA) equipped with three transducers: a high-frequency linear-array 15–6 MHz (HFL50xp), a curved-array abdominal 5–2 MHz (C60xp) and a phased-array cardiac 5–1 MHz (P21xp) transducer. The footprints of the transducers are 5 cm, 6 cm, and 2.1 cm, respectively.

For the VATS procedure, isolated ventilation of the dependent lung via a double-lumen endotracheal tube was necessary. First, all patients underwent general anesthesia, were intubated and ventilated, and placed in a lateral decubitus position. The ventilator was set to deliver a tidal volume of 5 ml kg^−1^ at a rate of 20 min^−1^. The anesthesiologist verified the position and depth of the double-lumen endotracheal tube with fiberoptic bronchoscopy.

**Fig. 1:**
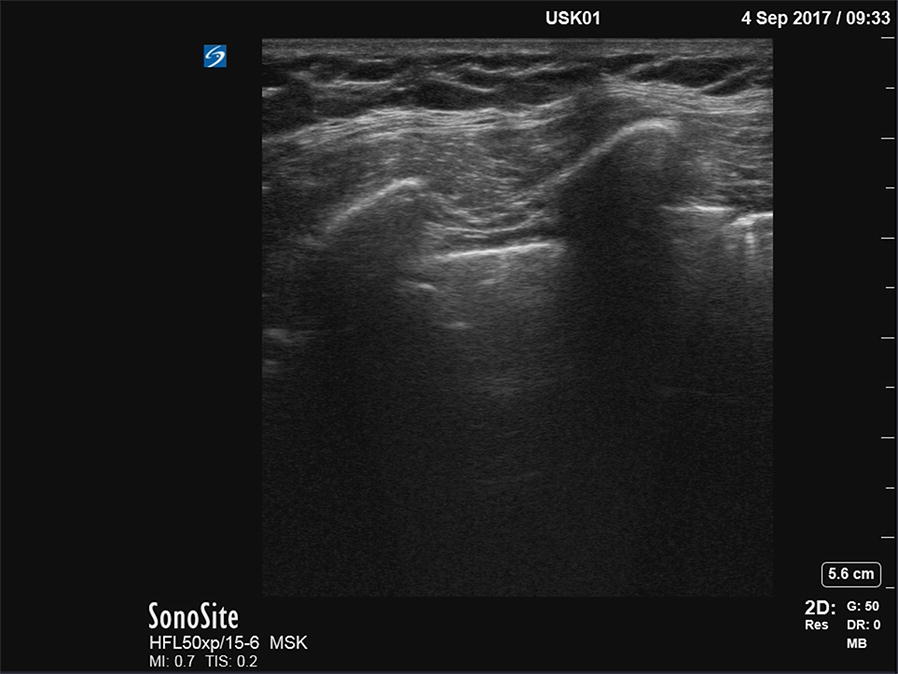
A typical uncropped image of the pleural interface, acquired with the phased-array transducer. On the right, the cropped version is displayed as is was played back to the observers

Second, the linear array, curved-array, and phased-array transducer were positioned over the fourth or fifth intercostal space at the axillary line in a craniocaudal orientation. 15-second ultrasound video clips were recorded of normal ventilation at a respiratory rate of 20 min^−1^. The zone of interest was the pleural line with its two adjacent ribs. A typical clip was framed as shown in Fig. 1.

**Fig. 2:**
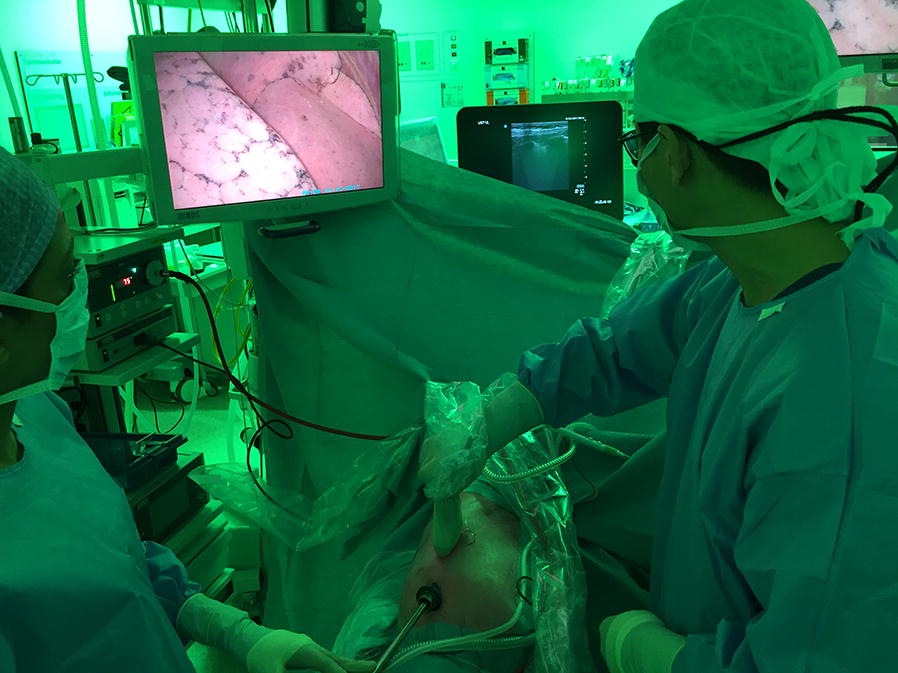
The surgeon performs lung ultrasonography in a patient with a confirmed pneumothorax and the videoscope in situ. The video screen displays an image of the inside of the right hemi-thorax and the collapsed right lung. The surgeon is handling the wrapped-up ultrasound transducer. The ultrasound device is shown in the back of the image

Third, after the chest was prepped and draped, ventilation of the non-dependent lung was interrupted while the surgeon opened the chest, introduced the videoscope and visually confirmed the collapse of the lung. Thereafter, the surgeon recorded three similar 15-s video clips of the established pneumothorax with the three transducers wrapped in sterile transducer covers (Fig. 2). Hence, six clips were recorded in every patient. The time interval between the induction of the pneumothorax (reference test) and the performance of the three ultrasound video clips was no longer than 2 min. No adverse events occurred.

**Fig. 3:**
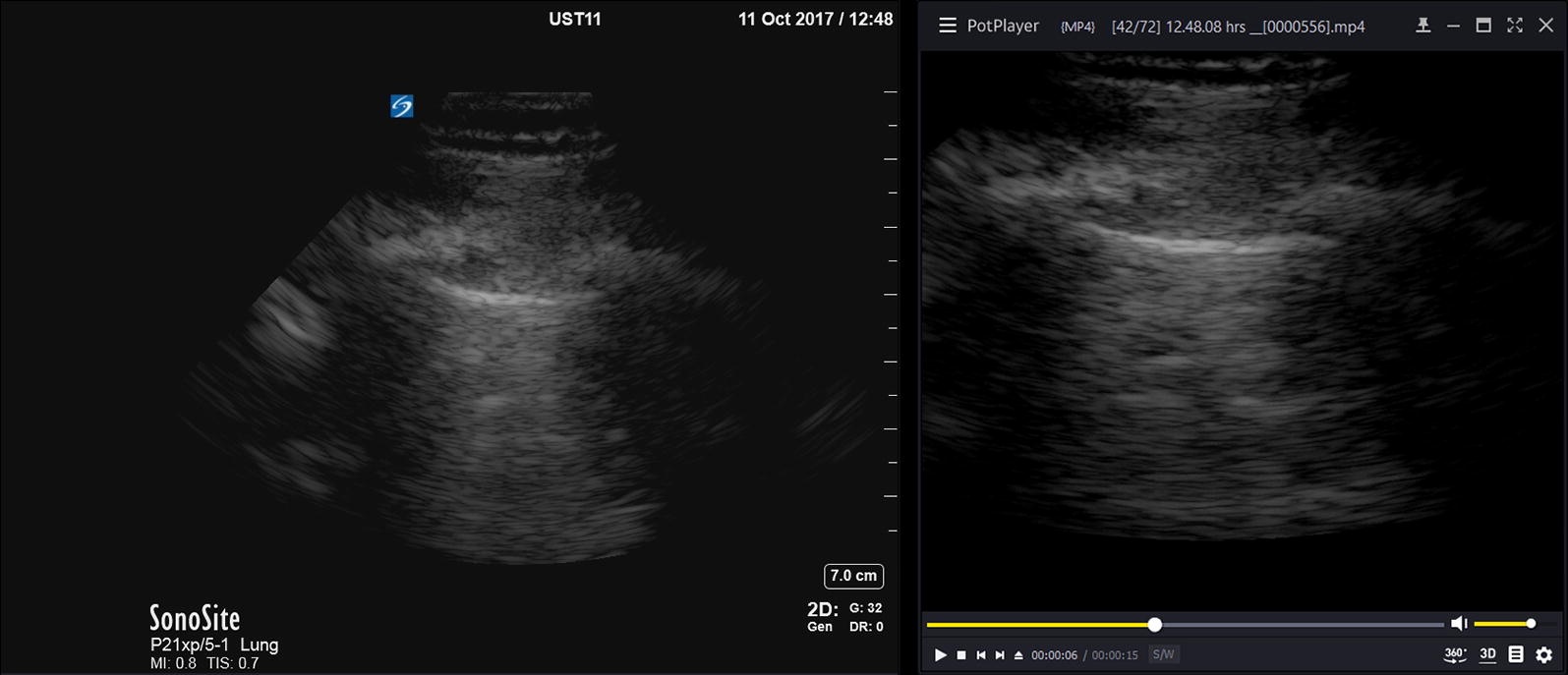
A typical uncropped and cropped image of the pleural interface, acquired with the phased-array transducer. On the right, the cropped version is displayed as it was played back to the observers

We cropped the video clips using iMovie for OS X, version 10.1.8 (Apple Inc., Cupertino, CA, USA). After we cropped and removed the text from the captured video clips, it was now no longer possible for the observers and the researchers to reliably recognize the transducer type by the image shape (rectangular or sector-shaped). An uncropped still image of the video clip and its cropped version are displayed in Fig. 3.

The Nijmegen physician-staffed HEMS carries a portable US machine since 2006. All HEMS physicians were trained in lung US either at the introduction of the US machine or at the start of their employment. They use lung US regularly in their prehospital practice.

**Fig. 4:**
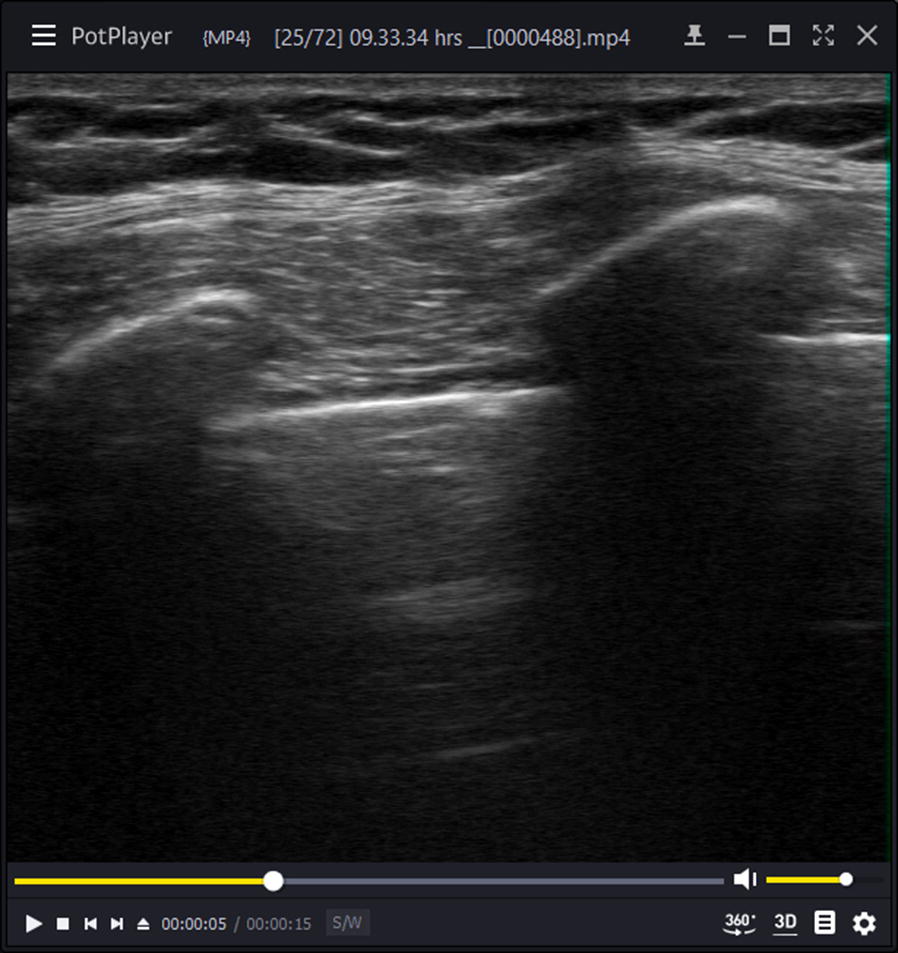
A cropped 15-s clip played in a random order to the observers

We recruited all 13 HEMS physicians (except the author, RK) and two anesthesiology residents with extensive experience in lung ultrasonography as observers to assess a randomized set of 66 15-s ultrasound clips. We used PotPlayer for Windows, version 1.7 (Kakao Corp., Jeju, South Korea) to separately randomize and playback the cropped set of clips for each observer (Fig. 4). Before the observers assessed the set of video clips, they were informed about how we acquired the clips and about the two possible conditions (normal ventilation and pneumothorax). Due to the cropping and randomization, the observers were blinded for the diagnosis and for the transducer type.

The observers were requested to pause the playback themselves when they were certain about the diagnosis based on the presence or lack of lung sliding, A-line sign, or B-lines. The equally blinded researcher recorded the elapsed time (s), the observer’s diagnosis and perceived image quality rated on a 1–5 Likert scale (1, very poor; 5, very good). For every observer, their experience (years) with lung US and preferred transducer type for lung US was recorded.

### Statistical analysis

Normally distributed data are reported as mean and standard deviation (SD). Data with an asymmetrical distribution are reported as median and interquartile range (IQR). We calculated the difference in elapsed time and image quality between transducers in every patient and observer: linear-array vs curved-array; linear-array vs phased-array; curved-array vs phased-array. Then, we fitted a linear mixed model with a random intercept to these differences to account for clustering within each observer. A two-tailed McNemar’s test for clustered data (Durkalski’s Chi-square test) was used to test for differences between the transducers in diagnostic performance. The Wilcoxon signed-rank test was used to test for differences in time until final diagnosis between diagnoses (normal ventilation and pneumothorax). For all statistical tests, significance level was set to .05. For statistical analysis, IBM SPSS Statistics for Windows, version 25.0 (IBM Corp., Armonk, NY, USA) and R, version 3.4.1, lme4 package installed (R Foundation for Statistical Computing, Vienna, Austria) were used.

## Results

### Patients

Sixty-six lung US video clips were acquired in 11 patients, of whom eight women, with a mean age of 64.0 years (± 9.03). Their mean weight was 66.1 kg (± 9.30) and the mean body mass index (BMI) was 24.3 kg m^−2^ (± 2.98). Surgery was performed on the left and right chest in four and seven cases, respectively. All participants had a pneumothorax after the surgeon opened the thorax.

### Observers

The video clips were observed by 15 physicians of whom 13 HEMS physicians (nine anesthesiologists, and four trauma surgeons) and two anesthesiology residents. These observers all had extensive experience (a mean of 7.1 years [± 3.58]) in lung ultrasonography.

Prior to the observations, six observers indicated to prefer a linear-array transducer for lung ultrasonography. Seven preferred a phased-array transducer and two had no preference. The curved-array transducer was preferred by none.

Each observer assessed the 66 cropped clips in a random order. There was no significant difference between their different backgrounds for success rate (correct or incorrect diagnosis) or time they needed to assess the video clips.

In 10 of the 990 judged clips, an observer could not decide on the diagnosis because the image quality was perceived to be too bad. Therefore, for data analysis where the diagnosis is a factor we used the data on 980 clips. The time was recorded from the start of video playback to the moment the observer declared to be unable to state a diagnose.

### Diagnostic performance

The overall sensitivity and specificity was 98.2% and 97.2%, positive predictive value (PPV) and negative predictive value (NPV) was 97.2% and 98.2%, respectively. A cross tabulation of the correct and incorrect diagnosis compared between transducers are displayed in Table 1.

**Table 1:** Cross tabulation of the number of correct and incorrect diagnoses compared between transducers

	Diagnosis	Correct	Incorrect	Total
		Curved-array transducer		
Linear-array transducer	Correct	313	7	320
	Incorrect	8	2	10
Total		321	9	330
		Phased-array transducer		
Linear-array transducer	Correct	308	12	320
	Incorrect	8	2	10
Total		316	14	330
		Phased-array transducer		
Curved-array transducer	Correct	309	12	321
	Incorrect	7	2	9
Total		316	14	330

The diagnostic performance measures for the different transducers for pneumothorax were calculated for 980 assessed clips and are displayed in Table 2.

**Table 2:** Diagnostic performance of the three ultrasound transducers for pneumothorax

	Linear-array transducer (%)	Curved-array transducer (%)	Phased-array transducer (%)	All transducers combined (%)
Sensitivity	97.5	98.2	98.8	98.2
Specificity	97.6	96.4	97.5	97.2

McNemar’s test for clustered data showed no significant difference in diagnostic performance between the different transducers. (Linear- vs curved-array: *p* = .706, linear- vs phased-array: *p* = .537, curved- vs phased-array: *p* = .515).

### Time

**Fig. 5:**
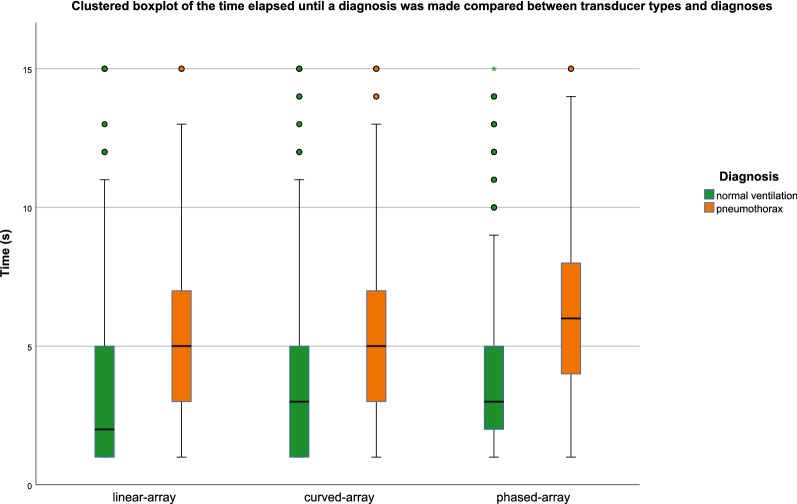
Boxplot of the elapsed time until a diagnosis was made compared between transducer types and diagnoses. The elapsed time until a diagnosis was stated by the observers. The time is represented in median seconds. The box represents the 25–75% interquartile range. The whiskers indicate the 95% confidence interval. There is a significant difference in the elapsed time until a diagnosis was made between normal ventilation and pneumothorax within all three transducers (*p* < .0001)

The time the observers needed to reach a diagnosis is displayed in Table 3 and Fig. 5.

**Table 3:** Time elapsed until a final diagnosis was made

Diagnosis	Linear-array transducer	Curved-array transducer	Phased-array transducer	All transducers combined
Normal ventilation	2 (1–5)	3 (1–5.5)	3 (2–5.5)	3 (1–5)
Pneumothorax	5 (3–7)	5 (3–7)	6 (3.5–8.5)	5 (3–7)
All diagnoses^a^	4 (2–6)	4 (2–6)	4 (2–7)	4 (2–6.25)

The data are presented as median seconds (interquartile range)

^a^This includes the ten clips without diagnosis

After we fitted the linear mixed model, we found a significant difference between the times that elapsed until a final diagnosis was made. Whit the linear-array transducer the diagnosis was made .51 s (*p* = .031) faster compared with the phased-array transducer. The curved-array transducer was .15 s (*p* = .049) faster than the phased-array transducer. We found no significant difference between the linear- and curved-array transducers. These comparisons between transducers are displayed in Table 4.

**Table 4:** Difference in time elapsed until a diagnosis was made between transducer types

Compared transducers	Estimate [95% CI]	*p* value
Linear-array vs curved-array	− .35 [− .78, .07]	.105
Linear-array vs phased-array	− .51 [− .97, − .05]	.031
Curved-array vs phased-array	− .15 [− .59, .28]	.049

This table presents the differences in elapsed time until a diagnosis was made between a combination of two transducers, using a linear mixed model with a random intercept

The differences are presented in seconds

A negative value indicates that less time elapsed using the left of the two compared transducers

Normal ventilation was diagnosed significantly faster than the diagnosis of a pneumothorax, regardless of transducer type. The Wilcoxon signed-rank test showed a significant difference overall (*p* < .0001) and within the three transducer groups as shown in Fig. 5 (*p* < .0001 in all three groups).

### Image quality

**Fig. 6:**
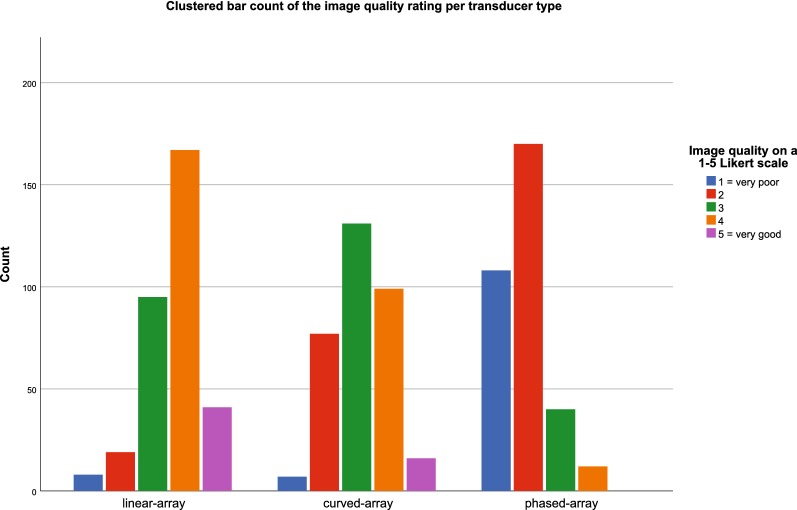
Clustered bar count of the image quality rating per transducer type. The Wilcoxon signed-rank test showed a significant difference in image quality between all three transducers (*p* < .0001)

Image quality was scored on a 5-point Likert scale. The image quality of the linear-, curved-, and phased-array transducers was appreciated at a median of 4 (IQR 3–4), 3 (IQR 2–4); 2 (IQR 1–2), respectively. Overall image quality was rated a median of 3 (IQR 2–4). The distribution of the image quality scores per transducer type is displayed in Fig. 6.

The Wilcoxon signed-rank test showed a significant difference in image quality between all three transducers (*p* < .0001).

After we fitted the linear mixed model, we found significant differences in image quality between all three transducers. The image quality with the linear-array transducer was 1.78 higher than the image quality with the phased-array transducer on a 5-point Likert scale. These comparisons in image quality between transducers are displayed in Table 5.

**Table 5:** Difference in image quality between transducer types

Compared transducers	Estimate [95% CI]	*p* value
Linear-array vs curved-array	.53 [.29, .76]	< .0001
Linear-array vs phased-array	1.78 [1.56, 2.01]	< .0001
Curved-array vs phased-array	1.25 [1.09, 1.42]	< .0001

This table presents the differences in reported image quality between a combination of two transducers, using a linear mixed model with a random intercept

The image quality was reported on a 5-point Likert scale: 1, very poor; 5, very good

A positive value indicates that the image quality was better with the left of the two compared transducers

The image quality was deemed too bad to make a diagnosis in ten cases: 8 of 330 phased-array transducer clips and 2 of 330 linear-array transducer clips. Of those, seven clips showed a pneumothorax and three showed normal lung sliding.

## Discussion

In this study, we found no difference in the diagnostic performance of the linear-array, curved-array, and phased-array transducer. The diagnostic performance was very good: sensitivity, specificity, PPV, and NPV were all between 96.4 and 98.8%. The observers needed an additional .51 s to reach a final diagnosis with the phased-array transducer compared to the linear-array transducer. A final diagnosis was reached much faster when lung sliding was present regardless of the transducer type.

The image quality scored by the observers on a 1–5 scale was significantly different between all three transducers; the linear-array transducer achieved the best scores, the phased-array transducer the worst. Moreover, the image quality was too bad to reach a diagnosis in eight phased-array transducer clips and two linear-array transducer clips.

These findings suggest that the actual diagnostic performance of the three transducers for pneumothorax is comparable. However, these experienced observers perceived the best image quality and needed the least amount of time when they judged the linear-array transducer clips. Based on these findings, the linear-array transducer might qualify as the preferred transducer for lung ultrasonography. However, the transducer choice may depend on more important factors such as the intended gamut of indications US is used for and whether the machine will be equipped with one or more transducers. In a single transducer setup, the best choice is probably a curved-array or a phased-array transducer to be able to evaluate both the abdomen and pericardium, in addition to the chest.

To our knowledge, there are no studies that have compared US transducers for diagnosing pneumothorax in a similar study design.

In a study with a comparable design, but not focused on pneumothorax, the authors compared a 10–5 MHz and a 14–5 MHz linear-array transducer for a wide array of emergency department point-of-care ultrasound indications [15]. However, lung ultrasonography was discussed only briefly. Overall, their observers most frequently preferred the 10–5 MHz transducer over the 14–5 MHz transducer.

In another study, the investigators compared a 5–10 MHz linear-array and a 1–5 MHz phased-array sector transducer in 55 patients scheduled for a thoracic-computed tomography (CT) scan [16]. The authors evaluated the diagnostic performance for pneumothorax, pleural effusion, consolidation, and interstitial syndrome. In six patients with a pneumothorax, confirmed with CT, they found that the linear-array transducer performed best with a sensitivity and specificity of 83% and 100%, respectively. The phased-array transducer showed a sensitivity and specificity of 67% and 100%. Sensitivity of both auscultation and chest radiography was only 50%. In our study, the gold standard was a thoracoscopically induced and confirmed pneumothorax. Because we assessed 495 ultrasound clips showing pneumothorax, the diagnostic performance we found is more reliable.

We hypothesized that the linear-array transducer would have the best diagnostic performance. This study, however, showed no difference in diagnostic accuracy between the transducers.

The Nijmegen HEMS introduced prehospital ultrasonography to the Netherlands in 2006 and used a phased-array transducer ever since. Only years later, a linear-array transducer was added. A curved-array transducer has never been used. This history might explain the transducer preferences of the observers and the high and equal diagnostic performance between transducers.

Although diagnostic performance is equal, we recommend the linear-array transducer for (prehospital) lung ultrasonography. The diagnosis is made faster and with a better image quality. These are important advantages in the dynamic prehospital environment, HEMS physicians encounter challenges such as time pressure, limited working space, residual clothing, defibrillator pads, and Velcro^®^ straps. Most importantly, the interpretation of US images may be hampered by sunlight or precipitation. When the HEMS physicians have the best possible image quality, they can better deal with these factors and do the best possible for our patients.

Furthermore, when the linear-array transducer is installed as the default transducer, it may have additional advantages. It is the preferred transducer for vascular access and assessment of the airway and endotracheal tube position [15]. These matters often take precedence over detailed assessment of breathing, although it may be of vital importance to be informed about a significant pneumothorax before airway management is commenced.

In addition, the linear-array transducer is superior for ultrasound-guided regional anesthesia (UGRA) in severely injured or trapped extremities and for optic nerve sheath diameter (ONSD) measurements in traumatic brain injury (TBI) patients [17]. For abdominal ultrasound and echocardiography, however, the phased-array or curved-array transducer is still invaluable.

The observers were able to successfully assess the video clips of normal ventilation and pneumothorax without having access to the US machine or the patient. This situation is comparable to a telemedicine setup in which the US operator could be at a different physical location than the observer of the images. Therefore, we agree that lung ultrasound can be successfully used in telemedicine setups [18].

### Strengths and limitations

We chose a unique approach to select VATS patients with a freshly induced and visually confirmed pneumothorax as the gold standard. In addition, we included the video clips of the same patients with normal anatomy before surgery. Another unique aspect was the cropping of the video clips thus transducers could not be identified by any text or image or sector shape.

A limitation of this study is that it might be underpowered because we could not demonstrate a difference in diagnostic performance between transducers. It could also mean the difference is close to none.

Another limitation is that we informed the observers that all patients were ventilated similarly and that besides a pneumothorax in half of the video clips, no other pathology was present. This could be an advantage for them judging the clips and might have resulted in an overestimation of the diagnostic performance and time needed. The performance could have been even better when we acquired M-mode clips looking for lung pulse or clips that included the lung point [14].

Conversely, most observers were uncomfortable with the fact that they had to assess video clips and that they were therefore unable to reposition or adjust the transducer, adjust the gain or depth, or compare with the contralateral chest. In addition, it was regarded a disadvantage that no additional clinical parameters were provided. The setting of pulmonary surgery introduced some minor challenges. In some clips, lung sliding was minimal, probably due to the lung-protective ventilator settings. B-lines were still present, obviously. In contrast to the normal clips, the pneumothorax clips were recorded with the transducer wrapped in a sterile cover. In theory, this might result in a slightly degraded US image.

Image quality might be overstated in video clips in which the diagnosis was made fast and perceived to be easy. Those clips might be scored good quality because they were ‘easy’ to assess.

A suggestion for future studies comparing ultrasound transducers might be to include subjects of all BMIs to better represent the general population of critically ill and injured patients.

## Conclusion

In conclusion, we found no difference in the effectiveness of detecting or excluding a pneumothorax between a high-frequency linear-array ultrasound transducer, a curved-array, and a phased-array transducer. Besides many indications for which it is essential, the linear-array transducer produces the best image quality in lung ultrasonography. Based only on image quality, the linear-array transducer might qualify as the preferred transducer for lung ultrasonography and the preferred default in our prehospital setting.

## Abbreviations

CT: computed-tomography
IQR: interquartile range
HEMS: helicopter emergency medical service
NPV: negative predictive value
ONSD: optic nerve sheath diameter
PPV: positive predictive value
SD: standard deviation
TBI: traumatic brain injury
UGRA: ultrasound-guided regional anesthesia
US: ultrasonography
VATS: video-assisted thoracoscopic surgery
